# Butyrate producing microbiota are reduced in chronic kidney diseases

**DOI:** 10.1038/s41598-021-02865-0

**Published:** 2021-12-07

**Authors:** Bei Gao, Adarsh Jose, Norma Alonzo-Palma, Taimur Malik, Divya Shankaranarayanan, Renu Regunathan-Shenk, Dominic S. Raj

**Affiliations:** 1 School of Marine Sciences, Nanjing University of Information Science and Technology, Nanjing, 210044 China; 2 Kaleido Biosciences, Lexington, MA USA; 3 Division of Kidney Diseases and Hypertension, George Washington University School of Medicine, 2150 Pennsylvania Ave NW, Washington, DC 20037 USA

**Keywords:** Microbiome, Microbial ecology

## Abstract

Chronic kidney disease is a major public health concern that affects millions of people globally. Alterations in gut microbiota composition have been observed in patients with chronic kidney disease. Nevertheless, the correlation between the gut microbiota and disease severity has not been investigated. In this study, we performed shot-gun metagenomics sequencing and identified several taxonomic and functional signatures associated with disease severity in patients with chronic kidney disease. We noted that 19 microbial genera were significantly associated with the severity of chronic kidney disease. The butyrate-producing bacteria were reduced in patients with advanced stages of chronic kidney diseases. In addition, functional metagenomics showed that two-component systems, metabolic activity and regulation of co-factor were significantly associated with the disease severity. Our study provides valuable information for the development of microbiota-oriented therapeutic strategies for chronic kidney disease.

## Introduction

Trillions of microorganisms are residing in the human gastrointestinal tract which influence the host physiology, nutrition, metabolism and immune function. The gut microbiota has vast metabolic capacity. Microbial metabolites, such as short-chain fatty acids, bile acids and trimethylamine N-oxide (TMAO), play an essential role in human health and diseases. As the breakdown products of complex carbohydrates, short-chain fatty acids are important energy sources for colonocytes, which also enhance gut barrier function and regulate regulatory T cell homeostasis in the colon^1,2^. The gut microbiota composition is influenced by many factors, including fiber intake, use of antibiotics and phosphate binders, colonic transit time, urea concentration, gastrointestinal uric acid and oxalate secretion^3^.

Chronic kidney disease (CKD) is a global public health burden. With persistent abnormalities in kidney structure and function, chronic kidney disease affects 8 to 16% of people worldwide^4^. In advanced CKD, the colon becomes the main route of uric acid and oxalate excretion. High concentrations of blood urea also reach the colon through gastrointestinal secretions. The high concentration of urea and uric acid causes overgrowth of bacteria containing urease and uricase^5^. In addition, colonic transit time is decreased in uremic patients, and the rate of constipation is 63% in hemodialysis patients and 29% in peritoneal dialysis patients^6^. These changes in chronic kidney disease affect the living environment of the gut microbiota.

Altered gut microbiota composition has been reported in patients with CKD, including those with end-stage renal disease (ESRD), compared with healthy controls^7,8^. The differing microbial population has also been shown in peritoneal dialysis patients as compared to healthy controls^9^. However, these findings only focused on the taxonomic alterations by either 16S rRNA sequencing or real-time PCR. Functional consequences of the changes in the gut microbiota composition were not investigated. In addition, the dysregulation of gut microbiota in different stages in CKD is understudied. Therefore, this study was designed to determine the shift in microbial community composition and assess the alteration in microbial functional capacity in CKD patients with different disease severity.

## Results

### Patients

Stool samples from a total of 52 patients with varying stages of CKD were collected in this study: CKD3A (n = 12), CKD3B (n = 11), CKD4 (n = 15), CKD5 (n = 4) and ESRD (n = 10) (Table 1). Patients’ characteristics are summarized in Table 1. Among 52 patients, 31 were reported to have Type 2 diabetes mellitus and 7 patients were reported to have human immunodeficiency virus (HIV) infection. As expected, urine protein creatinine ratio, serum creatinine and blood urea nitrogen level increased with progressing stages of CKD (CKD 3A to ESRD). There was no significant difference in fat, protein, carbohydrates, dietary fiber and calorie intake between CKD patients with different stages (Supplementary Table S1).

**Table 1:** Patients’ characteristics.

	CKD 3A	CKD 3B	CKD 4	CKD 5	ESRD
Patient number	12	11	15	4	10
Male	7 (58.3%)	2 (18.2%)	10 (66.7%)	2 (50.0%)	8 (80.0%)
Human immunodeficiency virus (HIV) infection	1 (8.3%)	1 (9.1%)	1 (6.7%)	0 (0.0%)	4 (40.0%)
Diabetes	9 (75.0%)	5 (45.5%)	10 (66.7%)	2 (50.0%)	5 (50.0%)
Body mass index (BMI)	30.97 (22.82–49.06)	29.23 (21.91–40.85)	31.99 (21.25–61.43)	31.8 (25.65–36.77)	26.88 (18.01–42.56)
Urine protein creatinine ratio (mg/g)	13.10 (4.20–397.30)	21.50 (6.00–930.00)	680.60 (3.50–3598.60)	1718.00 (170.90–1975.10)	675.80 (155.00–2451.60)
Diastolic blood pressure	97.75 (97.30–98.80)	97.75 (97.40–98.50)	97.90 (97.00–98.70)	97.90 (97.80–98.00)	97.80 (96.50–98.40)
Systolic blood pressure	129.25 (113.00–152.00)	125.00 (109.50–158.00)	135.00 (95.00–188.00)	137.5 (115.50–165.00)	148 (123.00–176.00)
Blood urea nitrogen (mg/dL)	19 (12–26)	26 (14–32)	40 (27–59)	48.5 (31–63)	53 (34–84)
Serum creatinine (mg/dL)	1.43 (1.07–1.68)	1.60 (1.41–2.39)	2.73 (1.84–4.27)	6.85 (4.46–7.56)	10.81 (5.76–16.86)
Potassium (mmol/L)	4.40 (3.20–5.00)	4.50 (3.40–103.00)	4.70 (3.10–5.30)	4.25 (4.00–5.50)	4.95 (3.70–6.80)
Sodium (mmol/L)	140.50 (137.00–143.00)	142.00 (139.00–147.00)	140.00 (137.00–147.00)	141.50 (139.00–147.00)	139.00 (133.00–143.00)

### Alpha and beta-diversity

**Figure 1:**
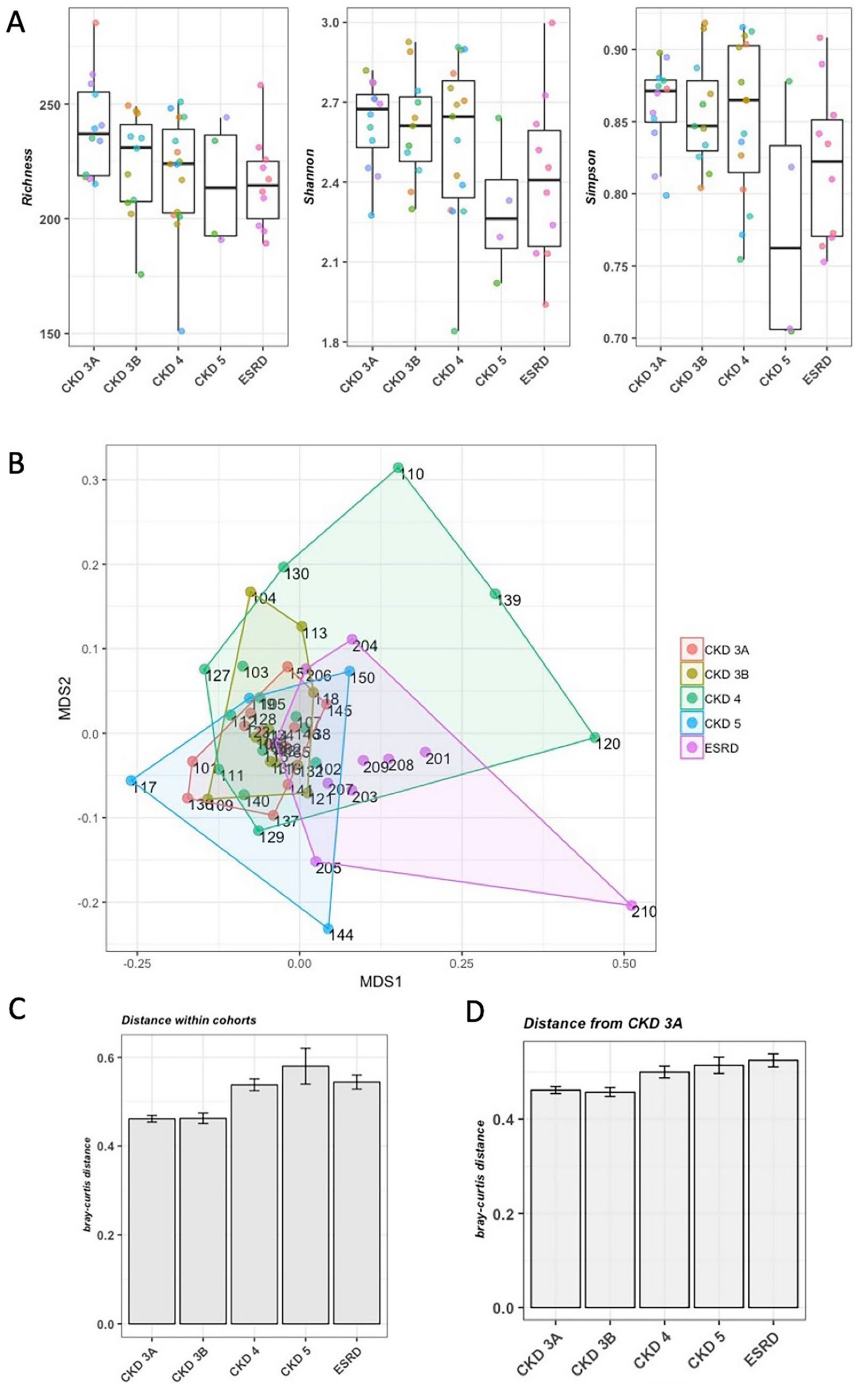
Alpha and beta diversity. (**A**) Richness, Shannon and Simpson index. (**B**) Ordination plot using Bray–Curtis dissimilarity score. Adonis test p-value = 0.057. (**C**) Mean Bray–Curtis distance within the cohorts. P-values were calculated by ANOVA following by Tukey post-hoc test. CKD 3B vs. CKD 3A p = 0.99; CKD 4 vs. CKD 3A p < 0.001; CKD 5 vs. CKD 3A p < 0.001; ESRD vs. CKD 3A p < 0.01; CKD 4 vs. CKD 3B p < 0.001; CKD 5 vs. CKD 3B p < 0.001; ESRD vs. CKD 3B p < 0.01; CKD 5 vs. CKD 4 p = 0.36; ESRD vs. CKD 4 p = 0.97; ESRD vs. CKD 5 p = 0.56. (**D**) Mean Bray–Curtis distance from the earliest stage CKD (CKD 3A).

Richness and Shannon index were not significantly different between different patient groups, meanwhile the CKD5 group showed a significant decrease in Simpson diversity compared with CKD 3A (FDR < 0.06), CKD3B (FDR < 0.06) and CKD4 (FDR < 0.07) groups (Fig. 1A). The variation within patient groups was greater than variation across groups, and the overall gut microbiota composition of different CKD patient groups was not statistically different (Adonis test p-value = 0.057, Fig. 1B) even though ESRD patients tended to group separately from the patients with earlier stage patients (CKD 3A/3B, Fig. 1B). Mean Bray–Curtis distance within the cohorts was shown to be higher in the later stages of CKD (CDK 4/5 and ESRD) when compared with samples from subjects with early stage disease (CKD 3A/3B) (Fig. 1C). Mean Bray–Curtis distance of samples from the earliest stage group (CKD 3A) showed that the microbial composition of the subjects in the ESRD group is farthest away from patients in CKD 3A stage (Fig. 1D). Spearman correlation analysis showed that the dissimilarity in BMI between the subjects did not show any significant correlation with the taxonomic dissimilarity between the subjects (p-value = 0.182, Supplementary Fig. S1). In addition, the HIV status of the subjects did not have any significant effect on the taxonomic dissimilarity (ANOVA FDR > 0.19, Supplementary Fig. S2).

### Gut microbiota composition

**Figure 2:**
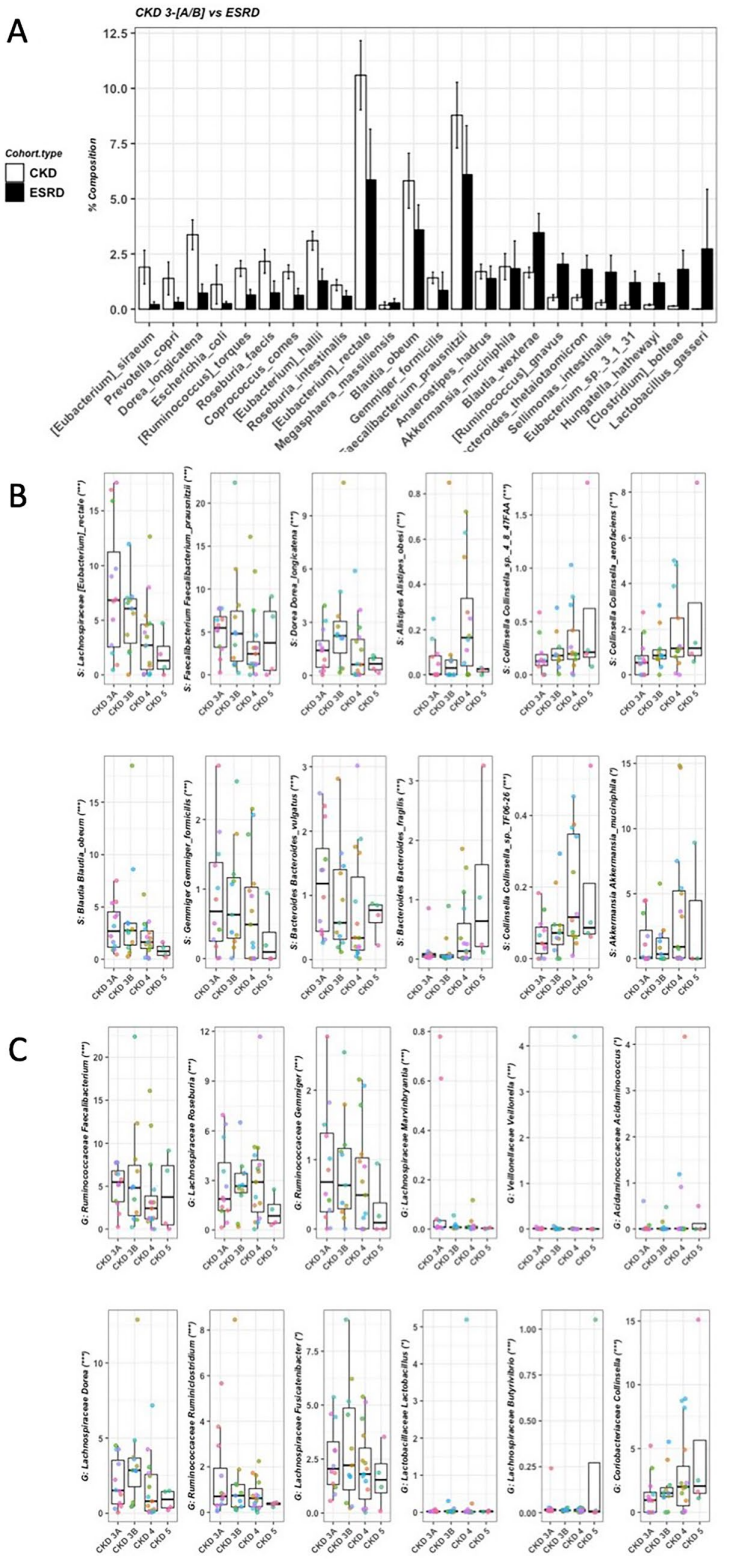
Microbial composition. (**A**) Significant microbial species between early stage CKD (n = 23) and ESRD patients (n = 10). Wilcox-rank sum test was used to calculate p-values and multiple testing was corrected using FDR. Relative abundance of microbial species > 1% in one of the subjects and with FDR < 0.1 were shown. (**B**) Microbial species significantly associated with the severity of CKD. Association was calculated by Maaslin2. Top six species showing negative and positive association with disease severity were shown. (**C**) Microbial genera significantly associated with the severity of CKD. Association was calculated by Maaslin2. Top six species showing negative and positive association with disease severity were shown. ***FDR < 0.0005; **FDR < 0.005; *FDR < 0.05.

A total of 24 microbial species were significantly different between early stage patients (CKD 3A/B) and ESRD patients (FDR < 0.1, Fig. 2A). A total of 57 microbial species were significantly associated with the disease severity (Supplementary Table S2). The top six positive and negative associations are shown in Fig. 2B, among which *Eubacterium rectale* and species belonging to *Collinsealla* genera demonstrated the strongest association. At genus level, 19 microbial genera were significantly associated with the severity of CKD (Supplementary Table S3), and the top six positive and negative correlations are shown in Fig. 2C.

**Figure 3:**
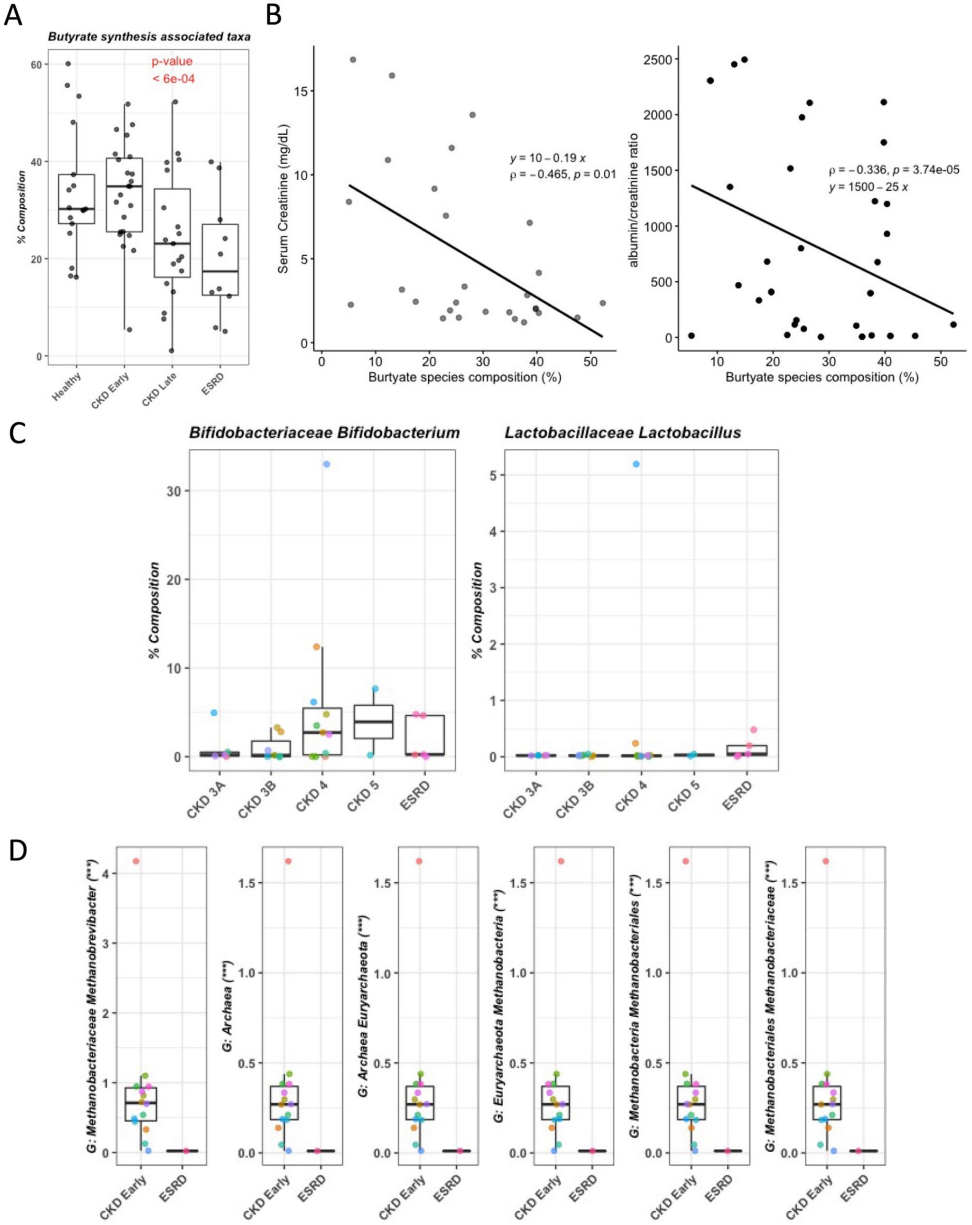
Key microbial taxa. (**A**) Butyrate synthesis associated taxa. P-value was calculated using linear mixed effect modelling. (**B**) The serum creatinine level shows statistically significant (p-value = 0.01) negative association with butyrate synthesis associated species composition (Left). The albumin/creatinine ratio in the serum shows statistically significant (p-value = 3.74e−5) negative association with butyrate synthesis associated species composition (Right). The spearman correlation was used for estimating association. (**C**) *Bifidobacterium* and *Lactobacillus*. (**D**) *Methanobacteria*. ***FDR < 0.0005; **FDR < 0.005; *FDR < 0.05.

Further, we identified 22 microbial species harboring genes encoding butyrate kinase and butyryl-coA transferase in patients. The butyrate synthesis associated species composition was estimated by summing the relative composition of 22 known butyrate synthesis species identified in the subjects at > 1% relative composition. The summed up relative composition from these species represents the butyrate synthesis capacity. The Linear mixed effect model showed the butyrate synthesis associated taxa was negatively associated with the disease severity (p-value = 6e^−04^) and ESRD patients had the lowest level of butyrate synthesis associated taxa (Fig. 3A). Spearman correlation analysis showed that the serum creatinine levels were significantly correlated with butyrate synthesis associated species composition (Fig. 3B, Left). The albumin/creatinine ratio in the serum showed significantly negative association with butyrate synthesis associated species composition (Fig. 3B, Right). The level of *Bifidobacterium* was increased with disease severity, meanwhile *lactobacillus* was decreased with disease severity (Fig. 3C). Interestingly, *Methanobacteria* increased from early to late stage CKD patients, but it was not detected in patients with ESRD (Fig. 3D).

**Figure 4:**
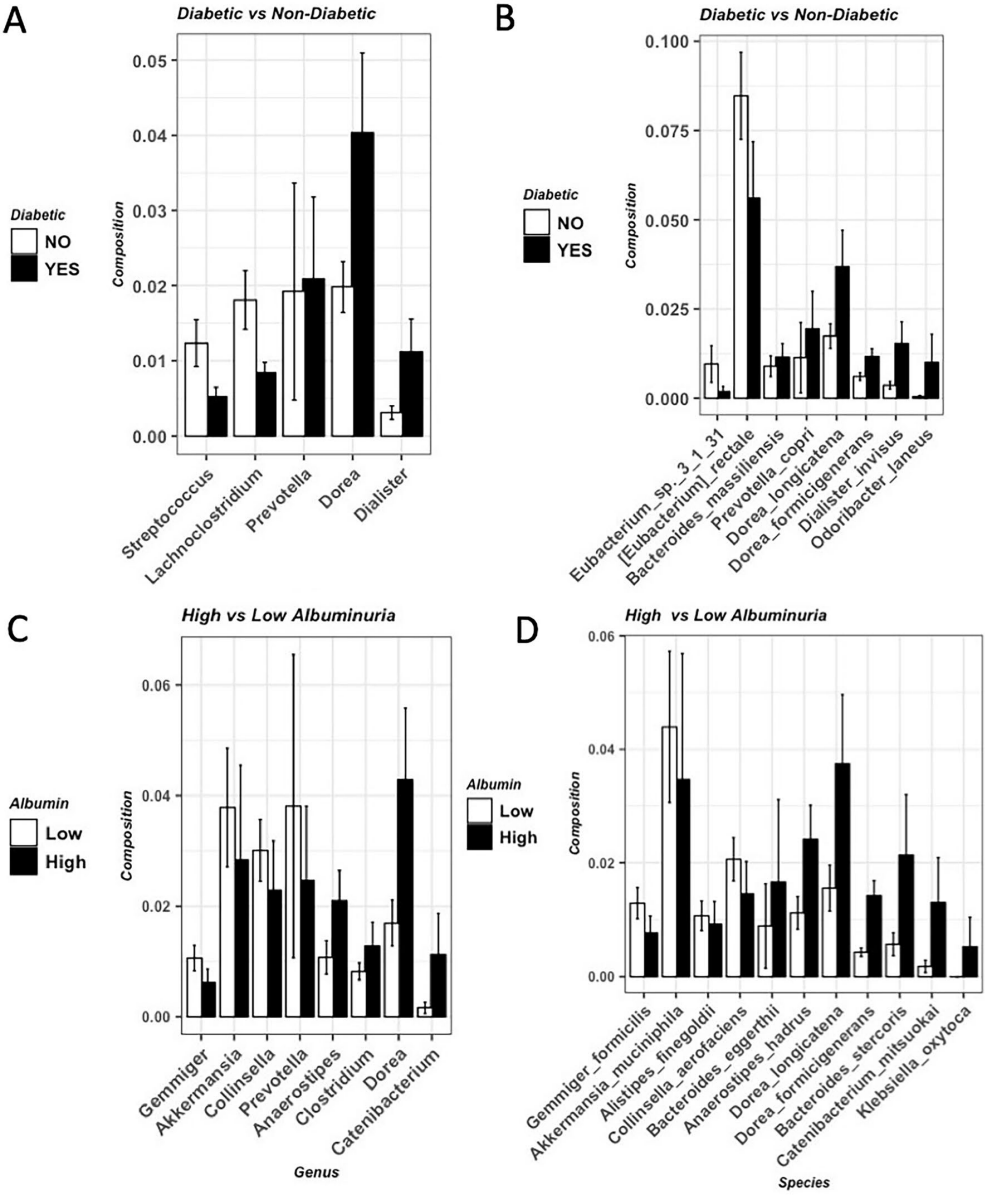
Different microbial species between different groups. (**A**) Comparison between diabetic patients and non-diabetic patients at genera level. (**B**) Comparison between diabetic patients and non-diabetic patients at species level. (**C**) Comparison between patients with high albuminuria and low albuminuria at genera level. (**D**) Comparison between patients with high albuminuria and low albuminuria at species level.

Next, we identified five microbial genera and eight microbial species that were different between the diabetic patients and non-diabetic patients (FDR < 0.01, Fig. 4A,B). However, the diabetic status didn’t significantly affect the gut microbial community structure (Adonis test p-value > 0.874, Supplementary Fig. S3). Eight microbial genera and 11 microbial species were different between patients with high (albumin/creatinine ratio > 300) and low (albumin/creatinine ratio < 30) albuminuria (FDR < 0.01, Fig. 4C,D).

### Functional metagenome

**Figure 5:**
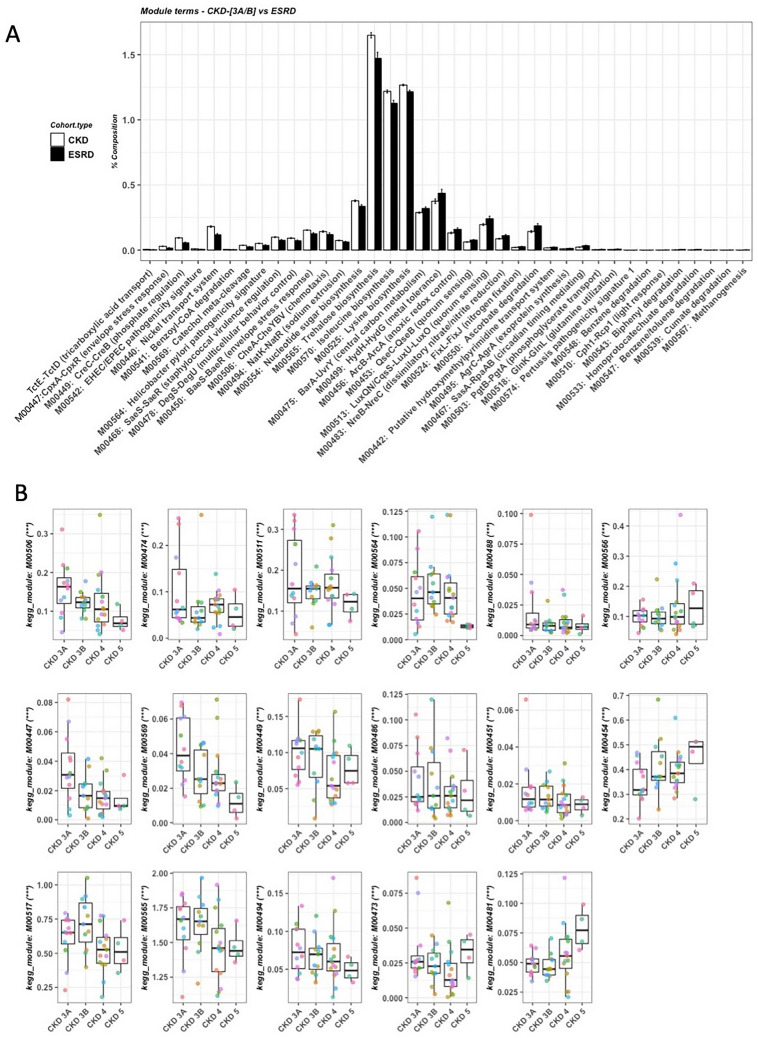
KEGG modules. (**A**) Significant KEGG modules between early stage CKD (n = 23) and ESRD patients (n = 10). Wilcox-rank sum test was used to calculate p-values of top 100 modules and multiple testing was corrected using FDR (FDR < 0.05 were shown). (**B**) KEGG modules significantly associated with the severity of CKD (FDR < 0.05). Association was calculated by Maaslin2. KEGG definitions: Supplementary Table S4. ***FDR < 0.0005; **FDR < 0.005; *FDR < 0.05.

**Figure 6:**
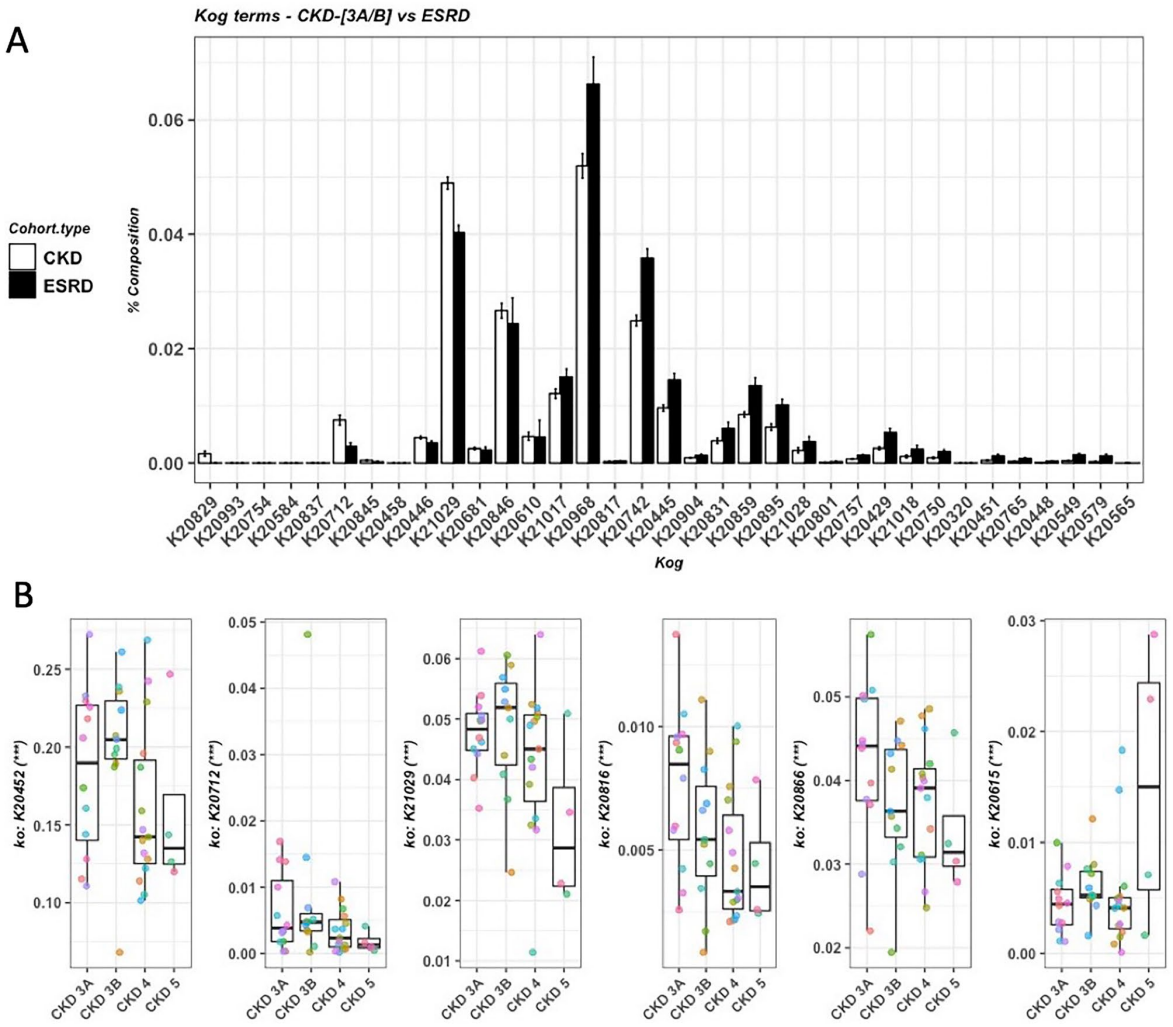
KOG enzymes. (**A**) Significant KOG enzymes between early stage CKD 3A/B (n = 23) and ESRD patients (n = 10). Wilcox-rank sum test was used to calculate p-values of top 100 KOG enzymes and multiple testing was corrected using FDR (FDR < 0.05 were shown). KOG definitions: Supplementary Table S5. (**B**) KOG families significantly associated with the severity of CKD (FDR < 0.05). Association was calculated by Maaslin2. K20712 3-(hydroxyamino)phenol mutase; K21029 molybdopterin-synthase adenylyltransferase; K20816 streptothricin hydrolase; K20615 capreomycidine synthase; K20866 glucose-1-phosphatase; K20452 dimethylmaleate hydratase large subunit. ***FDR < 0.0005; **FDR < 0.005; *FDR < 0.05.

A total of 38 Kyoto Encyclopedia of Genes and Genomes (KEGG) modules were different between early stage patients (CKD 3A/B) compared with ESRD patients (FDR < 0.1, Fig. 5A). Significant association was found between 17 KEGG modules and the disease severity (FDR < 0.1, Fig. 5B, Supplementary Table S4), among which 14 modules were negatively correlated with disease severity, while the remainder were positively correlated with the disease severity including dipeptide transport system and two-component regulatory systems involved in cell wall stress response and potassium transport. A total of 35 Cluster of Orthologous groups (KOG) enzymes were significantly different between early stage patients (CKD 3A/B) compared with ESRD patients (FDR < 0.1, Fig. 6A, Supplementary Table S5). Significant association was found between six KOG enzymes and the disease severity (FDR < 0.1, Fig. 6B, Supplementary Table S6), among which five were negatively correlated with the disease severity and capreomycidine synthase was positively correlated with the disease severity.

### Stool butyrate level

**Figure 7:**
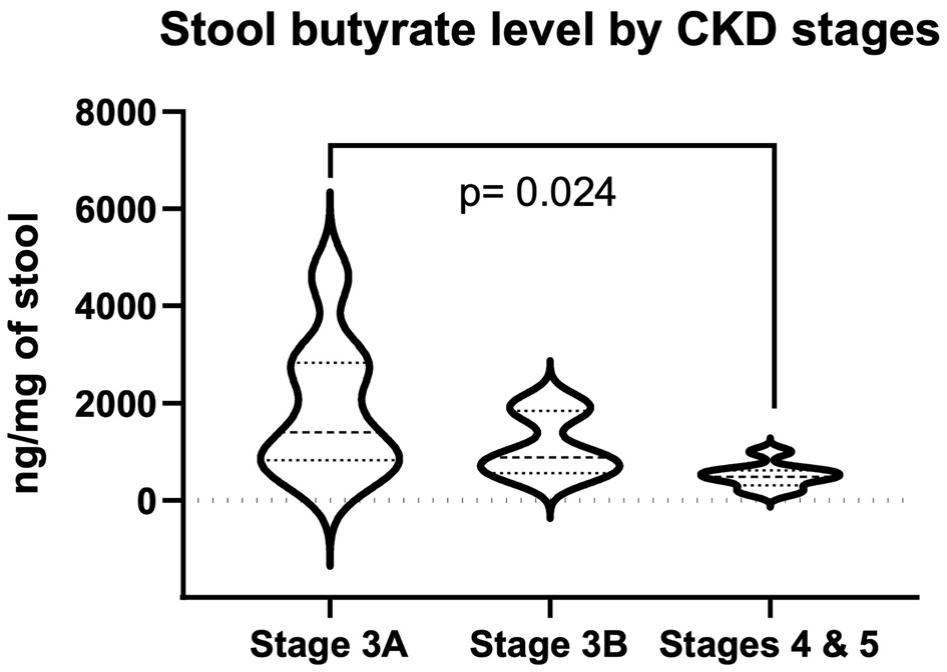
Stool butyrate level in CKD patients. Overall p-value was calculated using one-way ANOVA (p-value = 0.0240). Adjusted p-value was calculated using Tukey’s multiple comparison test (Stage 3A vs. Stage 3B adjusted p-value = 0.2962; Stage 3A vs. Stage 4 and 5 adjusted p-value = 0.0216; Stage 3B vs. Stage 4 and 5 adjusted p-value = 0.4743).

To confirm the impact of butyrate synthesis associated species on butyrate production, we measured the butyrate concentration in stool samples. Due to the sample availability, stool samples were collected from 8 patients with stage 3A, 6 patients with stage 3B, and 8 patients with stage 4 and 5. Stool butyrate level was significantly reduced in CDK patients with stage 4 and 5 compared with patients with stage 3A (Fig. 7, ANOVA overall p-value = 0.024, Tukey’s multiple comparison test adjusted p-value = 0.0216).

## Discussion

Using shot-gun metagenomics, we identified microbial taxa and functional signatures associated with the disease severity in patients with CKD. Notably, butyrate producers reduced with disease severity (Fig. 3A). Butyrate is a four-carbon short-chain fatty acid, produced by the microbial digestion of dietary fibers. Butyrate is a major energy source for colonocytes^10^. Butyrate interacts with G protein-coupled receptors (GPCRs) and acts as an inhibitor for histone deacetylase (HDAC). Butyrate has anti-inflammatory effects, enhances intestinal barrier function and mucosal immunity^11^. The decrease in butyrate producers in advanced stage of CKD might have adverse effects on the host. In addition, *Methanobacteria* increased from early to late stage CKD patients (Fig. 3D). We have demonstrated that intestinal colonization with methanogenic Archaea lowers the Trimethylamine *N*-oxide level in plasma, with a tendency to attenuate the atherosclerosis burden in Apoe^−/−^mice^12^. Several *Collinsella* species were positively associated with the CKD disease severity (Supplementary Table S2). Increased abundance of *Collinsella* has been reported to be associated with increased cholesterol levels in healthy individuals^13^. A reduced level of *Collinsella* by a structured weight loss program were reported in obese type 2 diabetics and lower abundance of *Collinsella* maintained over longer time periods may decrease the susceptibility for cardiovascular disease^14^.

Bacteria sense, respond and adapt to the changes and stimuli in their environment through two-component systems, which contain a sensor protein-histidine kinase for receiving external input signals and a response regulator that conveys a proper change in the bacterial cell physiology^15^. Among the 17 significant KEGG modules associated with disease severity, 13 were two-component regulatory systems (Fig. 5B). These two-component regulatory systems were involved in stress response (CpxA-CpxR, LiaS-LiaR), antimicrobial peptide resistance (BasS-BasR), capsule synthesis (RcsC-RcsD-RcsB), chemotaxis (CheA-CheYBV), signaling (RpfC-RpfG), cell fate control (PleC-PleD), metabolism (CitA-CitB, DcuS-DcuR, UhpB-UhpA), and regulation of phosphate, sodium and potassium (CreC-CreB, KdpD-KdpE, NatK-NatR). These changes in the two-component systems suggest that the gut microbiota respond and adapt to the changes in the environmental niches when early stage CKD progressed to ESRD.

The metabolic capacity of the gut microbiota also differed when CKD progressed to advanced stage. Four KEGG module involved in different compound metabolism were negatively correlated with the disease severity, including hexose phosphate uptake, trehalose biosynthesis, citrate fermentation and catechol meta-cleavage, one of the major pathways for the degradation of aromatic compounds. In addition, among five KEGG enzymes negatively correlated with the disease severity, three were involved in the metabolism, including 3-(hydroxyamino)phenol mutase, glucose-1-phosphatase and dimethylmaleate hydratase large subunit. 3-(hydroxyamino)phenol mutase is involved in the degradative pathway of 3-nitrophenol, a phenolic compound. Glucose-1-phosphatase primarily acts as a scavenger for glucose^16^. Dimethylmaleate hydratase catalyzes the conversion of (2R,3S)-2,3-dimethylmalate to dimethylmaleate^17^, which is a dimethyl ester of maleic acid.

In addition to the metabolic activity, the gut microbiota’s functional capacity of cofactor biosynthesis and regulation of antibiotics were also affected by the disease severity. Molybdopterin-synthase adenylyltransferase and streptothricin hydrolase were negatively correlated with the disease severity (Fig. 6B). Molybdopterin-synthase adenylyltransferase participates in the synthesis of molybdopterins, which are a class of cofactors found in a large group of enzymes. Streptothricin hydrolase catalyzes the hydrolysis of streptolidine lactam group in streptothricin antibiotics and inactivate them^18^. Capreomycidine synthase was positively associated with the disease severity (Fig. 6B), which catalyzes the biosynthesis of capreomycidine, a nonproteinogenic amino acid which is involved in the biosynthesis of the tuberactinomycin class of peptide antibiotics such as viomycin and capreomycin.

There are some limitations of this study. The sample size is relatively small, and the study population is heterogeneous with relatively small numbers of patients in each CKD stage. Thus, the findings from this study need to be validated in a larger cohort. Treatment information was not available in this patient cohort, which could influence the intestinal microbiota. Although we found the metabolic capacity of the gut microbiota altered with the disease severity, its impact on the host metabolism needs to be further validated using metabolomics approaches. In addition, further experiments are needed to investigate the causal relationship between the change of gut microbiota and CKD, such as fecal microbiota transplantations. In conclusion, this study provides valuable information about the association between disease severity and the dysregulation of the gut microbiota composition and its functional capacity. This knowledge can be valuable in the development of gut microbiota targeted precision medicine for patients with different stages of CKD.

## Patients and methods

### Study participants

Participants were enrolled from the outpatient clinic at the George Washington University. The major inclusion criteria were confirmed diagnosis of stable CKD and age ≥ 18 year. The major exclusion criteria were use of prebiotics, probiotics, or antibiotics during the previous 8 weeks; current infection, inflammatory bowel disease, chronic diarrhea, acute kidney injury and immunosuppressive therapy. Dietary assessment using the Block Food Frequency Questionnaire (https://nutritionquest.com) was administered by the experienced coordinators at George Washington University^19^. The study was approved by the George Washington University School of Medicine review boards and the study was conducted in accordance with the Declaration of Helsinki. Informed consent was obtained from all study participants.

Stool samples were collected by the study participants, stored at 4 °C, and transported in Styrofoam coolers with ice packs to the clinical research unit within 24 h. Blood and urine samples were collected by the research coordinators. Participants received compensation for study participation that was based, in part, on providing the stool samples.

### DNA sequencing of fecal bacterial cell pellets and microbiome analysis

The DNA of fecal bacterial pellets were extracted and sequenced at Diversigen using BoosterShot Shallow Shotgun Sequencing. A mean sequencing depth of ≥ 0.5 M reads were used per sample. Samples were extracted with MO Bio PowerFecal or MO Bio PowerSoil Pro (Qiagen) automated for high throughput on QiaCube (Qiagen), with bead beating in 0.1 mm glass bead plates. Samples were then quantified using Quant-iT Picogreen dsDNA Assay (Invitrogen). a procedure adapted from the Nextera Library Prep kit (Illumina) was then used for preparing the libraries. Libraries were sequenced either on an Illumina NextSeq using single-end 1 × 150 reads with a NextSeq 500/550 High Output v2 kit (Illumina). DNA sequences were first filtered for low quality (Q-Score < 30) and length (< 50) followed by trimming of adapter sequences using cutadapt^20^ software. Fastq files were then compiled into a single fasta using shi7. Sequences were then trimmed to a maximum length of 100 bp. The filtered and trimmed DNA sequences were then aligned to a database containing all representative genomes in RefSeq for bacteria with additional manually curated strains compiled by Diversigen. The reads were aligned at 97% identity against the reference genomes in the database. All sequences were compared to all reference sequence in the curated database using fully gapped alignment with BURST^21^. Ties were broken by minimizing the overall number of unique Operational Taxonomic Units (OTUs). Each input sequence was assigned the lowest common ancestor that was consistent across at least 80% of all reference sequences tied for best hit for taxonomy assignment. The OTU tables were rarified at 10,000 reads and samples with fewer than 10,000 reads were discarded. KEGG functional profiling was obtained by using the method similar to one previously described^22^. The profiles were summarized at KEGG Ortholog (KO) levels and KEGG Module levels.

### Stool butyrate measurement

Due to the sample availability, butyrate concentrations were measured in stool samples collected from 8 patients with stage 3A, 6 patients with stage 3B, and 8 patients with stage 4 and 5. Stool butyrate measurement was performed at the West Coast Metabolomics Center following standard protocol.

### Statistical analysis

To evaluate effect on the overall microbiome structure, we square-root transformed relative abundances of bacterial species and calculated pair-wise bray–curtis dissimilarities between the subjects. We then performed principal coordinate analysis on the dissimilarity matrix. Further, we performed Wilcoxon rank test for identifying significantly different taxa between (1) early stage CKD (3A, 3B) and ESRD patients, (2) diabetic and non-diabetic patients and (3) high and low albuminuria patients. The test results for all comparisons were corrected using FDR. Only taxa with relative abundances greater than or equal to 0.1% in either group were examined. The same approach was used to identify significantly different KEGG modules and KO families between (1) early stage CKD (3A, 3B) and ESRD patients. We used a heuristic scoring scheme for representing the severity of the subjects. CKD 3A subjects were assigned a severity score of 10, CKD 3B subjects were assigned 20, CKD 4 subjects were assigned 30, CKD 5 stage subjects were assigned 40 and ESRD subjects were assigned a score of 50. MaAsLin2 was then used to explore the effect of disease severity on the microbial relative abundance and functional composition of the microbial metagenome^23^. FDR < 0.1 was considered as significant.

## Supplementary Information

Supplementary Information.
