# Supplementary material for: Butyrate producing microbiota are reduced in chronic kidney diseases
Source: Sci Rep. 2021 Dec 7;11:23530. doi: 10.1038/s41598-021-02865-0 (PMC8651744; doi:10.1038/s41598-021-02865-0)
Supplement: Supplementary file 1 — Supplementary Information. [file 41598_2021_2865_MOESM1_ESM.docx]

**Butyrate Producing Microbiota are Reduced in Chronic Kidney Diseases**

Bei Gao^1^, Adarsh Jose^2^, Norma Alonzo-Palma^2^, Taimur Malik^3^, Rohan Paul^3^, Divya Shankaranarayanan^3^, Renu Regunathan-Shenk^3^, Dominic Raj^3^*

**Affiliations:**

*^1^ School of Marine Sciences, Nanjing University of Information Science and Technology, Nanjing, China*

*^2^ Kaleido Biosciences, Lexington, Massachusetts, USA*

*^3^ Division of Kidney Diseases and Hypertension, George Washington University School of Medicine, Washington DC, USA*

*** Address for communication**

Dominic S Raj, MD

Professor and Chief

Division of kidney Diseases and Hypertension

George Washington University

2150 Pennsylvania Ave NW

Washington DC

Email: draj@mfa.gwu.edu

Phone 202 741 2283

**Table of Contents**

**Table S1.** Patients’ dietary pattern ………………………………………………………………………3

**Table S2.** Microbial species associated with disease severity………………………………………..3

**Table S3.** Microbial genera associated with disease severity…………………………………………4

**Table S4**. KEGG modules associated with disease severity…………………………………………..5

**Table S5.** Significant KOG enzymes in ESRD compared with early stage patients (CKD 3A/B)….5

**Table S6.** KOG enzymes associated with disease severity……………………………………………6

**Figure S1.** The dissimilarity in BMI between the subjects did not show any significant correlation with the taxonomic dissimilarity between the subjects…………………………….7

**Figure S2**. The HIV status of the subjects did not have any significant effect on the taxonomic dissimilarity…………………………………………………………………………………….8

**Figure S3.** The ordination plot of subjects labeled by their status of diabetes...………………9

**Table S1.** Patients’ dietary pattern

|  | 3A(n=12) | 3B(n=11) | 4(n=15) | 5(n=4) | ESRD(n=10) | p-value |
| --- | --- | --- | --- | --- | --- | --- |
| Calorie Intake (kCal) | 1195.78 ± 641.68 | 1335.72 ± 718.74 | 1178.99 ± 733.81 | 857.44 ± 447.2 | 1345.07 ± 493.77 | 0.45 |
| Fats (g) | 56.38 ± 31.3 | 67.12 ± 33.35 | 59.09 ± 40.03 | 36.58 ± 18.53 | 69.36 ± 24.82 | 0.18 |
| Protein (g) | 49.8 ± 31.81 | 49.5 ± 26.75 | 50.52 ± 31.13 | 29.64 ± 16.09 | 55.46 ± 20.06 | 0.34 |
| Carbohydrates (g) | 129.12 ± 74.20 | 132.14 ± 80.82 | 121.75 ± 71.12 | 107.35 ± 62.58 | 128.99 ± 61.86 | 0.94 |
| Dietary Fiber (g) | 13.79 ± 9.75 | 10.86 ± 6.04 | 13.47 ± 7.92 | 10.41 ± 4.62 | 11.49 ± 6.55 | 0.58 |

**Table S2.** Microbial species associated with disease severity

| **Species** | **Coefficient** | **Q.value** |
| --- | --- | --- |
| Lachnospiraceae [Eubacterium]_rectale | -0.007498557 | 3.598E-10 |
| Blautia Blautia_obeum | -0.003630685 | 1.86327E-07 |
| Faecalibacterium Faecalibacterium_prausnitzii | -0.003464261 | 0.001991567 |
| Gemmiger Gemmiger_formicilis | -0.001605978 | 0.005181657 |
| Dorea Dorea_longicatena | -0.001582253 | 0.031786483 |
| Bacteroides Bacteroides_vulgatus | -0.001551695 | 0.009581624 |
| Ruminiclostridium [Eubacterium]_siraeum | -0.001091135 | 0.057433187 |
| Roseburia Roseburia_hominis | -0.001085436 | 2.60617E-05 |
| Blautia [Ruminococcus]_torques | -0.001038925 | 0.076387547 |
| Roseburia Roseburia_intestinalis | -0.000967098 | 0.029733616 |
| Alistipes Alistipes_putredinis | -0.000966972 | 0.076387547 |
| Dorea Dorea_formicigenerans | -0.000900029 | 0.013427912 |
| Monoglobus Monoglobus_pectinilyticus | -0.000871964 | 3.01721E-05 |
| Eubacterium Eubacterium_ramulus | -0.000787515 | 0.008487745 |
| Neglecta Neglecta_timonensis | -0.000650662 | 0.098482134 |
| Pyramidobacter Pyramidobacter_piscolens | -0.000616742 | 0.007010512 |
| Blautia Blautia_hydrogenotrophica | -0.000540529 | 0.013427912 |
| Bacteria bacterium_LF-3 | -0.000503966 | 0.076387547 |
| Ruminococcus Ruminococcus_faecis | -0.000493804 | 0.022447671 |
| Blautia Blautia_sp._Marseille-P2398 | -0.000450721 | 0.022534693 |
| Eubacterium Eubacterium_ventriosum | -0.000407864 | 0.029733616 |
| Alistipes Alistipes_indistinctus | -0.000350154 | 0.017884987 |
| Streptococcus Streptococcus_salivarius | -0.000333737 | 0.006456292 |
| Anaeromassilibacillus Anaeromassilibacillus_sp._Marseille-P3371 | -0.000327443 | 0.003011363 |
| Bacteroides Bacteroides_eggerthii | -0.000299365 | 0.001746578 |
| Tyzzerella Tyzzerella_nexilis | -0.000266839 | 0.005176724 |
| Streptococcus Streptococcus_thermophilus | -0.000199165 | 0.083124717 |
| Alistipes Alistipes_onderdonkii | -0.000187865 | 0.076387547 |
| Negativibacillus Negativibacillus_massiliensis | -0.00018453 | 0.046555765 |
| Marvinbryantia Marvinbryantia_formatexigens | -0.000164255 | 0.000370913 |
| Coprobacillus Coprobacillus_sp._29_1 | -0.000157904 | 0.017672481 |
| Bacteroides Bacteroides_sp._4_3_47FAA | -0.000151394 | 0.031786483 |
| Eubacterium Eubacterium_sp._3_1_31 | -0.000148706 | 0.084536153 |
| Eisenbergiella Eisenbergiella_tayi | -0.000145785 | 0.074822804 |
| Lachnospiraceae Lachnospiraceae_bacterium_TF01-11 | -0.000126689 | 0.029733616 |
| Clostridium Clostridium_sp._L2-50 | -0.00012658 | 0.001572422 |
| Clostridium Clostridium_perfringens | -0.000110797 | 9.65241E-06 |
| Butyrivibrio Butyrivibrio_crossotus | -0.00011022 | 0.005181657 |
| Bacteroides Bacteroides_sp._HMSC067B03 | -0.000108421 | 0.076387547 |
| Coprococcus Coprococcus_eutactus | -0.000105992 | 0.072108079 |
| Streptococcus Streptococcus_pasteurianus | -9.7947E-05 | 6.64573E-05 |
| Bacteroides Bacteroides_fluxus | -8.25021E-05 | 0.009101961 |
| Bacteroides Bacteroides_salyersiae | -6.45735E-05 | 0.041228718 |
| Klebsiella Klebsiella_pneumoniae | -3.12046E-05 | 0.07388851 |
| Slackia Slackia_piriformis | 5.17296E-05 | 0.00016546 |
| Prevotella Prevotella_copri | 6.88345E-05 | 0.000610924 |
| Lactonifactor Lactonifactor_longoviformis | 7.0863E-05 | 0.024085772 |
| Lachnospiraceae Lachnospiraceae_bacterium_6_1_37FAA | 0.000103636 | 0.024085772 |
| Collinsella Collinsella_stercoris | 0.000104825 | 3.79635E-07 |
| Coprococcus Coprococcus_sp._HPP0048 | 0.000112321 | 0.022183793 |
| Bifidobacterium Bifidobacterium_pseudocatenulatum | 0.000135286 | 0.072446773 |
| Alistipes Alistipes_obesi | 0.000591918 | 0.022534693 |
| Bacteroides Bacteroides_fragilis | 0.00066456 | 0.028775915 |
| Collinsella Collinsella_sp._4_8_47FAA | 0.000770024 | 0.010464972 |
| Collinsella Collinsella_sp._TF06-26 | 0.001073811 | 3.4741E-07 |
| Collinsella Collinsella_aerofaciens | 0.001898412 | 0.004895457 |
| Akkermansia Akkermansia_muciniphila | 0.001973627 | 0.0870183 |

**Table S3.** Microbial genera associated with disease severity

| Genus | Coefficient | Q.value |
| --- | --- | --- |
| Ruminococcaceae Faecalibacterium | -0.003184949 | 0.001827677 |
| Lachnospiraceae Dorea | -0.00188715 | 0.008350222 |
| Lachnospiraceae Roseburia | -0.001684872 | 0.036979856 |
| Ruminococcaceae Ruminiclostridium | -0.001532318 | 0.00343541 |
| Ruminococcaceae Gemmiger | -0.001494582 | 0.00343541 |
| Lachnospiraceae Fusicatenibacter | -0.001451297 | 0.061985198 |
| Streptococcaceae Streptococcus | -0.00105503 | 0.00343541 |
| Clostridiales Monoglobus | -0.000780942 | 3.00901E-05 |
| Ruminococcaceae Neglecta | -0.000617746 | 0.082382503 |
| Synergistaceae Pyramidobacter | -0.000516054 | 0.005398277 |
| Ruminococcaceae Anaeromassilibacillus | -0.000332396 | 0.000122462 |
| Lachnospiraceae Tyzzerella | -0.000259975 | 0.001827677 |
| Ruminococcaceae Negativibacillus | -0.000176114 | 0.057666774 |
| Lachnospiraceae Marvinbryantia | -0.000164459 | 0.000129317 |
| Lactobacillaceae Lactobacillus | -0.00015294 | 0.067102713 |
| Veillonellaceae Veillonella | -0.000137628 | 0.002911821 |
| Lachnospiraceae Butyrivibrio | -0.000115065 | 0.00343541 |
| Acidaminococcaceae Acidaminococcus | 6.9299E-05 | 0.082382503 |
| Coriobacteriaceae Collinsella | 0.002591581 | 0.001827677 |

**Table S4**. KEGG modules associated with disease severity

| **KEGG Modules** | **Coefficient** | **Q.value** |
| --- | --- | --- |
| M00447 CpxA-CpxR (envelope stress response) two-component regulatory system | -3.20E-04 | 7.15E-10 |
| M00569 Catechol meta-cleavage, catechol => pyruvate + acetaldehyde | -2.74E-04 | 1.17E-09 |
| M00506 CheA-CheYBV (chemotaxis) two-component regulatory system | -3.24E-04 | 6.16E-05 |
| M00449 CreC-CreB (phosphate regulation) two-component regulatory system | -2.23E-04 | 2.43E-04 |
| M00481 LiaS-LiaR (cell wall stress response) two-component regulatory system | 1.36E-04 | 8.68E-04 |
| M00454 KdpD-KdpE (potassium transport) two-component regulatory system | 2.42E-04 | 8.68E-04 |
| M00565 Trehalose biosynthesis, D-glucose 1P => trehalose | -2.53E-04 | 2.15E-03 |
| M00474 RcsC-RcsD-RcsB (capsule synthesis) two-component regulatory system | -2.76E-04 | 2.47E-03 |
| M00488 DcuS-DcuR (aerobic C4-dicarboxylate metabolism) two-component regulatory system | -1.18E-04 | 2.47E-03 |
| M00451 BasS-BasR (antimicrobial peptide resistance) two-component regulatory system | -9.30E-05 | 2.68E-03 |
| M00494 NatK-NatR (sodium extrusion) two-component regulatory system | -1.65E-04 | 3.11E-03 |
| M00566 Dipeptide transport system, Firmicutes | 1.89E-04 | 9.93E-03 |
| M00511 PleC-PleD (cell fate control) two-component regulatory system | -2.34E-04 | 1.14E-02 |
| M00517 RpfC-RpfG (cell-to-cell signaling) two-component regulatory system | -3.08E-04 | 1.20E-02 |
| M00473 UhpB-UhpA (hexose phosphates uptake) two-component regulatory system | -1.24E-04 | 2.47E-02 |
| M00564 Helicobacter pylori pathogenicity signature, cagA pathogenicity island | -1.50E-04 | 3.04E-02 |
| M00486 CitA-CitB (citrate fermentation) two-component regulatory system | -1.49E-04 | 3.20E-02 |

**Table S5.** Significant KOG enzymes in ESRD compared with early stage patients (CKD 3A/B)

| **KOG ID** | **NAME** | **DEFINITION** | **Fold change** | **FDR** |
| --- | --- | --- | --- | --- |
| K20742 | ykfC | gamma-D-glutamyl-L-lysine dipeptidyl-peptidase [EC:3.4.14.13] | 5.27E-01 | 1.78E-05 |
| K20845 | txyA | endo-1,3-beta-xylanase [EC:3.2.1.32] | -1.28E+00 | 2.08E-05 |
| K20429 | vioA | dTDP-4-amino-4,6-dideoxy-D-glucose transaminase [EC:2.6.1.33] | 1.06E+00 | 5.50E-05 |
| K20829 | celS | cellulose 1,4-beta-cellobiosidase [EC:3.2.1.176] | -7.32E+00 | 5.50E-05 |
| K21029 | moeB | molybdopterin-synthase adenylyltransferase [EC:2.7.7.80] | -2.80E-01 | 1.57E-04 |
| K20757 | dthadh | threo-3-hydroxy-D-aspartate ammonia-lyase [EC:4.3.1.27] | 1.05E+00 | 2.43E-04 |
| K20549 | aci | 3,6-anhydro-L-galactonate cycloisomerase [EC:5.5.1.25] | 1.99E+00 | 3.54E-04 |
| K20837 | dnhA_B | 2,4-dinitroanisole O-demethylase [EC:3.3.2.14] | -1.42E+00 | 3.54E-04 |
| K20712 | glnA | 3-(hydroxyamino)phenol mutase [EC:5.4.4.3] | -1.37E+00 | 4.34E-04 |
| K20859 | phnPP | phosphoribosyl 1,2-cyclic phosphate 1,2-diphosphodiesterase [EC:3.1.4.57] | 6.77E-01 | 6.06E-04 |
| K20895 | ylmB | formylaminopyrimidine deformylase [EC:3.5.1.-] | 6.94E-01 | 8.03E-04 |
| K20750 | bapA | beta-peptidyl aminopeptidase [EC:3.4.11.25] | 1.20E+00 | 8.03E-04 |
| K20765 | camK | 6-oxocamphor hydrolase [EC:3.7.1.18] | 1.54E+00 | 8.03E-04 |
| K20801 | ltaA | L-allo-threonine aldolase [EC:4.1.2.49] | 9.81E-01 | 8.03E-04 |
| K20445 | ndhF | nicotinate dehydrogenase FAD-subunit [EC:1.17.1.5] | 5.95E-01 | 9.04E-04 |
| K20993 | estP | pyrethroid hydrolase [EC:3.1.1.88] | -5.65E+00 | 4.95E-03 |
| K20579 | tobZ | nebramycin 5' synthase [EC:6.1.2.2] | 2.55E+00 | 5.39E-03 |
| K20565 | neoG, neoQ | paromamine 6'-oxidase / 6'''-hydroxyneomycin C oxidase [EC:1.1.3.43 1.1.3.44] | Infinite* | 7.32E-03 |
| K20451 | mii | methylitaconate Delta-isomerase [EC:5.3.3.6] | 1.49E+00 | 7.73E-03 |
| K20610 | E4.2.2.25 | gellan lyase [EC:4.2.2.25] | -2.87E-02 | 8.29E-03 |
| K21028 | ynjE | molybdopterin synthase sulfurtransferase [EC:2.8.1.11] | 7.62E-01 | 9.91E-03 |
| K20458 | hcl | 3-hydroxybenzoate/4-hydroxybenzoate---CoA ligase [EC:6.2.1.37 6.2.1.27] | -9.50E-01 | 1.68E-02 |
| K20968 | exsA | AraC family transcriptional regulator, exoenzyme S synthesis regulatory protein ExsA | 3.52E-01 | 3.11E-02 |
| K20846 | cgkA | kappa-carrageenase [EC:3.2.1.83] | -1.30E-01 | 3.12E-02 |
| K20831 | ugl | gellan tetrasaccharide unsaturated glucuronyl hydrolase [EC:3.2.1.179] | 6.41E-01 | 3.12E-02 |
| K20754 | pstI | aqualysin 1 [EC:3.4.21.111] | -2.83E+00 | 3.12E-02 |
| K21018 | fumD | fumonisin B1 esterase [EC:3.1.1.87] | 1.09E+00 | 3.62E-02 |
| K20448 | ndhM | nicotinate dehydrogenase medium molybdopterin subunit [EC:1.17.1.5] | 1.73E+00 | 3.62E-02 |
| K20584 | livQ, parQ | 6'''-hydroxyparomomycin oxidase [EC:1.1.3.-] | -2.06E+00 | 3.62E-02 |

Note:

Fold change = mean of ESRD / mean of early stage CKD(3A/B)

*: only detected in ESRD patients

**Table S6.** KOG enzymes associated with disease severity

| **KOG ID** | NAME | DEFINITION | Coefficient | Q.value |
| --- | --- | --- | --- | --- |
| K20712 | glnA | 3-(hydroxyamino)phenol mutase [EC:5.4.4.3] | -1.23E-04 | 1.23E-04 |
| K21029 | moeB | molybdopterin-synthase adenylyltransferase [EC:2.7.7.80] | -1.05E-04 | 1.38E-04 |
| K20816 | sttH | streptothricin hydrolase [EC:3.5.2.19] | -7.23E-05 | 1.03E-03 |
| K20615 | vioD | capreomycidine synthase [EC:4.2.1.145] | 7.89E-05 | 5.67E-03 |
| K20866 | yihX | glucose-1-phosphatase [EC:3.1.3.10] | -5.63E-05 | 1.63E-02 |
| K20452 | dmdA | dimethylmaleate hydratase large subunit [EC:4.2.1.85] | -1.45E-04 | 1.82E-02 |

**
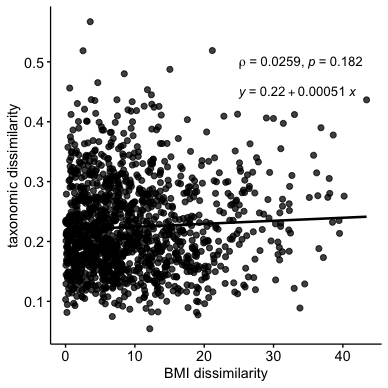
**

**Figure S1.** The dissimilarity in BMI between the subjects did not show any significant correlation with the taxonomic dissimilarity between the subjects (spearman correlation = 0.0259, p-value = 0.182). The bray-curtis distance between the subjects were estimated from the square root transformed microbial composition. The Euclidean distance metric was used to measure the BMI dissimilarity between the subjects.


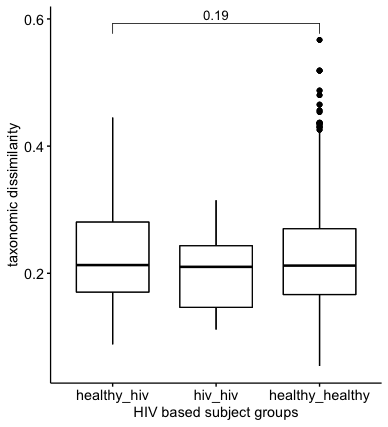


**Figure S2**. The HIV status of the subjects did not have any significant effect on the taxonomic dissimilarity (ANOVA FDR > 0.19). The bray-curtis distance between the subjects were estimated from the square root transformed microbial composition.


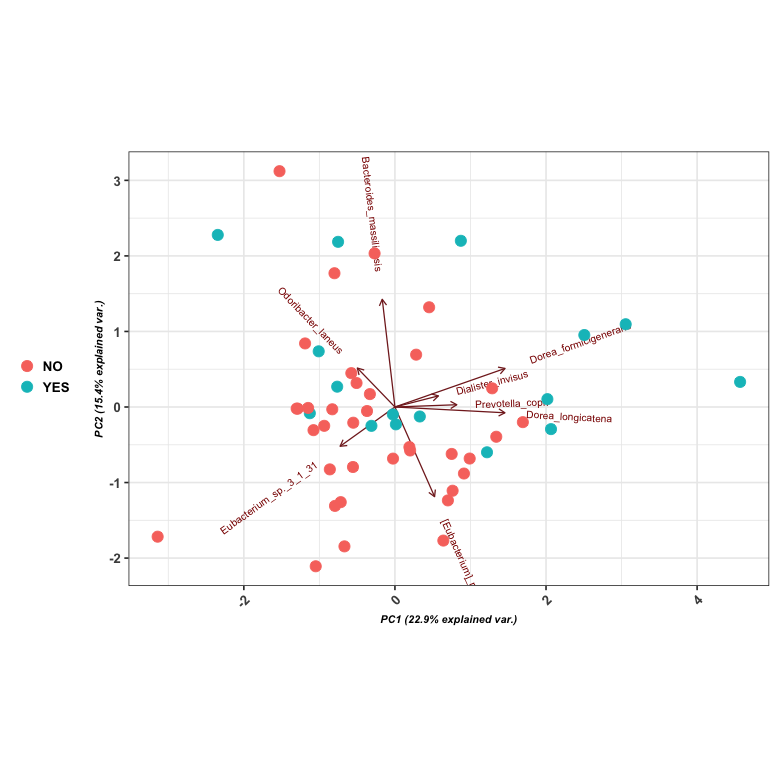


**Figure S3**. The ordination plot of subjects labeled by their status of diabetes. The ordination plot, is based on the relative composition of the 8 species identified to be different between diabetic and control subjects. The diabetic status didn’t significantly affect the gut microbial community structure (Adonis test p-value > 0.874). The adonis test was performed on the Bray-Curtis metric on the square transformed composition matrix factoring in the diabetic status of the subjects.
